# Two decades of prescription opioid-related cardiovascular safety signals: a FAERS-based pharmacovigilance study using machine learning

**DOI:** 10.3389/fdsfr.2026.1898403

**Published:** 2026-09-28

**Authors:** Li Ma, Johnna Berryhill, John Ihrie, Ru Chen, Beverly Lyn-Cook, Paul Rogers, Weigong Ge, Ningning Wu, Huixiao Hong, Weida Tong, Wen Zou

**Affiliations:** 1 Division of Bioinformatics and Biostatistics, National Center for Toxicological Research, U.S. FDA, Jefferson, AR, United States; 2 Department of Mathematics and Statistics, Arkansas State University, Jonesboro, AR, United States; 3 Division of Surveillance and Data Integration, Office of Surveillance Strategy and Risk Prioritization, Human Foods Program, U.S. FDA, College Park, MD, United States; 4 Office of New Drugs, Center for Drug Evaluation and Research, U.S. FDA, Silver Spring, MD, United States; 5 Division of Biochemistry and Toxicity, National Center for Toxicological Research, U.S. FDA, Jefferson, AR, United States; 6 Department of Information Science, University of Arkansas at Little Rock, Little Rock, AR, United States

**Keywords:** cardiovascular adverse events, data mining, FAERS, machine learning, prescription opioids

## Abstract

**Introduction:**

Prescription opioids are essential for pain management but have been increasingly linked to cardiovascular adverse events. Understanding these risks is critical for informed prescribing opioid drugs.

**Methods:**

Approximately 18 million adverse event reports from 2004 Quarter 1 to 2024 Quarter 3 in the U.S. FDA Adverse Event Reporting System (FAERS) were downloaded and processed. RxNorm was used to normalize prescription opioid drug names. Empirical Bayes Geometric Mean analysis was then performed to detect signals of disproportionate reporting. Cardiovascular-related Preferred Terms from MedDRA were applied to identify drug-adverse event pairs. Unsupervised machine learning approaches, such as network analysis, hierarchical clustering, and Pearson correlation analysis were employed to study cardiovascular risk relationships among the prescription opioids. R code for the correlation analysis was generated with the assistance of a large language model (Claude, Anthropic) and verified by the authors.

**Results:**

A total of 79,620 prescription opioid-cardiovascular adverse event pairs were identified, comprising 15 groups of FDA-approved prescription opioids and 291 unique cardiovascular adverse events. Fentanyl and oxycodone were the top two contributing drugs, while hypertension, hypotension, cardiac arrest, cardio-respiratory arrest, and tachycardia were the top five cardiovascular adverse events. The top 10 cardiovascular adverse events for each of the 15 groups of opioids were characterized. Network analysis and hierarchical clustering revealed close associations among fentanyl-remifentanil-sufentanil-morphine, hydrocodone-tramadol, and buprenorphine-oxymorphone-pentazocine based on shared cardiovascular adverse event profiles. The AI-assisted Pearson correlation heatmap further quantified pairwise profile similarities, identifying exceptionally strong correlations between pentazocine and buprenorphine (0.96), oxymorphone and methadone (r = 0.92), and between remifentanil and sufentanil (r = 0.91), and confirming the fentanyl-remifentanil-sufentanil-morphine cluster as the most internally coherent cardiovascular risk profiling group.

**Conclusion:**

This study provides the first large-scale, cardiovascular adverse events-focused pharmacovigilance analysis across FDA-approved prescription opioids using over two decades of FAERS data. The findings highlight the need for targeted cardiovascular risk assessment and monitoring, and support more refined prescribing practices and opioid safety labeling. The results showed that the integration of traditional Empirical Bayes signal detection with unsupervised machine learning may enable scalable pharmacovigilance analyses and uncover latent cardiovascular risk patterns that may be masked in broader adverse event assessments.

## 1 Introduction

Prescription opioids (POs) are one of the most frequently prescribed therapeutic drug classes, are considered the strongest analgesics, and have an important role in managing moderate to severe pain (NIDA, 2021). The opioid epidemic has been affecting communities and families in the US for decades and was declared a public health emergency in 2017 (U.S. Department of Health and Human Services, 2017). Besides overdose deaths, in 2022, nearly 9 million American reported misusing POs within the past year (CDC, 2024). The stark increase in opioid misuse and overdose deaths makes the current opioid epidemic a serious national crisis that affects public health as well as the social and economic welfare in the United States.

PO-related adverse events (AEs) encompassed a broad range of clinical outcomes and have been reported in the scientific literature (Andreaggi et al., 2020; Le et al., 2023; Gustafsson et al., 2024), underscoring the substantial public health burden associated with PO use. The most common AEs vary depending on the POs. Except for the risks such as nausea, constipation, dependence, respiratory depression, increased risk of falls, infections, and unintentional opioid overdose, etc. (Benyamin et al., 2008), POs may raise heart disease risk, especially with higher doses (Sung et al., 2024). POs have been implicated in the induction or worsening of cardiovascular diseases (CVDs), such as cardiac arrest (Ritter et al., 2022), myocardial infarction (Krantz et al., 2021), heart failure (Krantz et al., 2021) etc. Potential associations between several POs and cardiovascular adverse events (CVAEs) have been recognized and reported by several research groups (Khodneva et al., 2016; Chow et al., 2021; Cai et al., 2022; Liew et al., 2022). However, most of the studies used relatively small datasets for the retrospective cohort analyses or case reports and only restricted to medications or select patient groups. In addition, one or several POs were targeted in most of these studies. Therefore, these investigations did not provide a thorough and systematic evaluation of cardiovascular risks related to various kinds of POs in the context of real-world clinical practice. In addition, based on our limited knowledge, no investigations have been reported on the relationships of POs based on the developed CVAEs, although our previous investigations provided an outline of PO relations based on their whole AEs profiles (Le et al., 2023).

To compensate the gaps and comprehensively understand the cardiovascular risks associated with various PO use, the FDA Adverse Event Reporting System (FAERS) was utilized in this study for data mining to systematically evaluate and compare the CVAEs associated with various FDA-approved POs. The FAERS is considered as one of the largest valuable pharmacovigilance databases designed to monitor post-marketing drug safety (FDA, 2018a). It offers a rich source of real-world evidence, capturing spontaneous reports of suspected drug-associated AEs from healthcare professionals, patients, and manufacturers. However, the sheer volume, complexity, and variability of FAERS data also presented challenges including unstructured entry of drug names and report duplication, etc. Therefore, data mining techniques are commonly used to clean the data by standardizing the drug names, deduplicating rows, and identifying potential signals for drug safety assessment (FDA, 2018b; Zhao et al., 2021; Le et al., 2023; Le et al., 2024).

Recent advances in artificial intelligence (AI) and machine learning (ML) have enabled scalable mining of high-dimensional real-world data to uncover latent safety patterns that are difficult to detect using traditional hypothesis-driven approaches (Al-Nafjan et al., 2025; Rojas et al., 2025). In this study, FAERS was utilized to systematically investigate PO-associated CVAEs to systematically evaluate the CVD-related risk profiles of multiple POs and their associations. A similar approach was applied as our previous study (Le et al., 2023), but targeting on cardiovascular-related AEs only, thus providing more detailed identification of PO-related CVAEs which are of particular concern given their significant impact on public health. The results provided top ten CVAEs for each of 15 groups of FDA-approved POs and highlighted the most occurred CVAEs and the majority contributing POs. We then integrated machine learning and Pearson correlation visualization with traditional Empirical Bayes signal detection to characterize drug safety profiles and inter-drug relationships. The network analysis and hierarchical cluster analysis revealed the associations of the 15 groups of POs based on their CVAE profiles, and the associations showed different patterns compared to the previous study based on all AEs profiles. These results are expected to provide important information and knowledge to help the FDA drug reviewers, physicians, and patients be aware of potential cardiovascular risks to certain POs or combinations of opioids with other prescription drugs, therefore, preventing or reducing risks of the POs-associated CVD.

## 2 Materials and methods

### 2.1 Data retrieval and pre-processing

First, a list of POs was created using the FDA-approved drug list and the function “getApproximateMatch” of the RxNorm API (Le et al., 2023; Le et al., 2024; FDA, 2024). Instead of selecting the candidate with the highest matching score of RxNorm Concept Unique Identifier (RxCUI), we picked the description that matched with the RxNorm query the most. Subsequently, all AE reports from Quarter 1 of 2004 to Quarter 3 of 2024 in FAERS (FDA, 2024) were downloaded, and drug-AE pairs were extracted from the reports. The AEs in FAERS were coded as Preferred Terms (PTs) of the Medical Dictionary for Regulatory Activities (MedDRA) (ICH, 2024). The duplicated pairs were eliminated using a published approach (Jiang et al., 2024). All the PTs under the System Organ Class (SOC) cardiac disorders and vascular disorders of MedDRA were downloaded to identify CVAEs.

Drug name normalization is of paramount importance to the pharmacovigilant study. FAERS receives reports from a heterogeneous set of submitters, including but not limited to manufacturers, for whom reporting is mandatory under 21 CFR 314.80 (U.S. Food and Drug Administration, 1985), as well as health professionals and consumers, who report voluntarily through MedWatch (MedWatch Online Voluntary Reporting Form). Because the drug name field is captured as free text and is not validated against a controlled vocabulary, a single active ingredient may be reported under different forms, such as proprietary names, generic names, salt and ester variants, combination-product names, foreign trade names, and misspellings, etc. Therefore, consolidating these variants to a common identifier is a prerequisite for valid disproportionality analysis. In this study, the PO drug name normalization was pursued following our previously published approach (Le et al., 2024). In this method, RxCUIs of the drugs in the reports were retrieved using the same function “getApproximateMatch” of the RxNorm API. Then, a script written in Python 3.10 was implemented to retain the drug-AE pairs whose RxCUIs appeared in the PO reference list generated in the preceding step.

### 2.2 Potential safety signal detection

Disproportionality analysis methods are frequently employed to identify statistical associations between products and events in spontaneously reported adverse event databases, such as FAERS (FDA, 2018a). These methods are designed to compare the observed number of reports for a specific product-event combination or pair to an expected number, allowing for the detection of disproportionate reporting. There have been several studies quantitatively comparing the performance of popular disproportionality methods (Sakaeda et al., 2013; Hou et al., 2014; Candore et al., 2015; Noguchi et al., 2021), including the Proportional Reporting Ratio (PRR) (Evans et al., 2001), and the Empirical Bayes Geometric Mean (EBGM), etc (FDA, 2018b). The EBGM calculation is conceptually similar to the PRR but incorporates Bayesian “shrinkage” and stratification. This approach adjusts the disproportionality scores toward the null, particularly when the data includes small counts, thereby reducing the influence of spurious high values and minimizing false positives. The EBGM method offers a more stable estimate of the relative reporting rate of an event for a particular product compared to all other events in the database, and is known as the most conservative of popular signal detection algorithms (Szarfman et al., 2004; Sakaeda et al., 2014). The lower 95% confidence limit of the EBGM, referred to as EB05, provides a conservative estimate that is the primary metric used to evaluate signal strength; the upper 95% limit (EB95) is also reported, EB05 and EB95 together define a 90% credible interval around the EBGM.

In this project, EBGM scores were computed using the openEBGM package v0.9.1 (Canida and Ihrie, 2017) in R version 4.5.1 (R Core Team, 2026). Data squashing, as described in Canida and Ihrie (2017), was used to reduce computational burden. openEBGM’s “autoHyper ()” function was applied to estimate the optimal hyperparameters using a gradient-based approach. EBGM scores were calculated as well as their intervals. The potential safety signals were identified when the EB05 values were greater than one and the PO-AE pairs existed no less than three times, indicating disproportional reporting. The comparative analysis was then pursued on the identified potential safety signals.

### 2.3 Machine learning-enabled network and clustering analyses

The PO association network was constructed on the identified potential safety signals using the same approach described in previous work (Zhao et al., 2021; Le et al., 2023) to explore the relationships among the POs based on shared CVAEs. In this method, based on the constructed dataset of PO-CVAEs, the function “graph.constructor” in R package “igraph” v2.0.3 (Csardi, 2013) was utilized to create an unweighted and undirected bipartite graph to build the network of drug-adverse event associations, where nodes represented POs, and edges indicated the occurrence of a specific CVAE with a drug. The bipartite graph was then projected into a one-mode network of POs, focusing on PO-PO associations. This projection allowed for identification and analyzing of connections between POs that shared common CVAEs, reflecting their similarity in CVAE profiles. The resulting network was simplified by merging multiple edges between drug nodes and summing their weights, representing the total number of shared CVAEs. To enhance the visualization, edge widths were normalized according to their weights, and only the most significant edges (top 25% by weight) were labeled with their corresponding weights.

Hierarchical clustering was performed to examine the relationships between POs based on their CVAEs from a different perspective. A binary presence/absence matrix was first constructed, where each row represented a PO and each column represented a CVAE, with entries indicating the presence (1) or absence (0) of a CVAE for a particular PO. This matrix was used to calculate the Jaccard distance, a metric that quantifies dissimilarity between drug pairs based on the CVAEs they share. Hierarchical clustering was then applied using the average linkage method to group POs into clusters based on their Jaccard distances. The resulting dendrogram provided a visual representation of the clustering, allowing for the identification of distinct groups of POs with similar CVAE profiles.

### 2.4 Pearson correlation analysis

To further characterize pairwise similarities among the POs, a Pearson correlation matrix was computed across the counts of the SDR of all CVAEs for each PO. The authors designed the analysis, including selection of the Pearson correlation metric and its integration with Jaccard-distance hierarchical clustering. R code was generated with the assistance of Claude (claude-sonnet-4–5, Anthropic), reviewed and verified by the authors, and executed in R. The resulting clustered heatmap was visualized using the ComplexHeatmap package (Gu, 2022) in R. The authors take full responsibility for the accuracy and integrity of the results.

These analyses represent unsupervised machine learning approaches that identify latent similarity structures among prescription opioids based on their CVAE profiles, without imposing predefined risk categories or labels. The three approaches were chosen to identify the hidden associations among the 15 POs in different aspects: the bipartite network analysis identified the associations based on the shared CVAEs between each two-opioid pairs; Jaccard-based hierarchical clustering summarized the overall similarity of their binary presence (1)/absence (0) profiles; and the Pearson correlation quantified the linear concordance in the magnitude of SDR counts across PO-CVAEs, capturing intensity information that the binary methods discard. The Pearson correlation was selected because each PO’s CVAE profile was represented as a continuous vector of SDR counts, for which a linear association metric is appropriate and directly comparable across opioid pairs.

## 3 Results

### 3.1 Data retrieval and pre-processing

The flow chart of data retrieval and pre-processing was shown in Figure 1. Seventeen groups of FDA-approved POs were created. A total of 18,360,709 AE reports were retrieved and downloaded from FAERS from 2004, Quarter 1 to 2024, Quarter 3, consisting of 342,404,493 drug-AE pairs. Among those, 3,584,820 PO-AE pairs were then selected by filtering the drugs with 17 groups of FDA-approved POs. EBGM was then pursued on these pairs, and 1,851,856 pairs (51.66%) were identified as potential safety signals when the threshold was set up as EB05 greater than 1 and the PO-AE pairs were reported no less than 3 times. These signals were designated as signals of disproportional reports (SDRs, Table 1). After filtering with CVD-related PTs identified from MedDRA, 79,620 PO-CVAE SDRs were obtained, consisting of 15 unique FDA-approved POs and 291 unique CVAEs. The SDRs in this dataset were used for the following further analysis.

**FIGURE 1:**
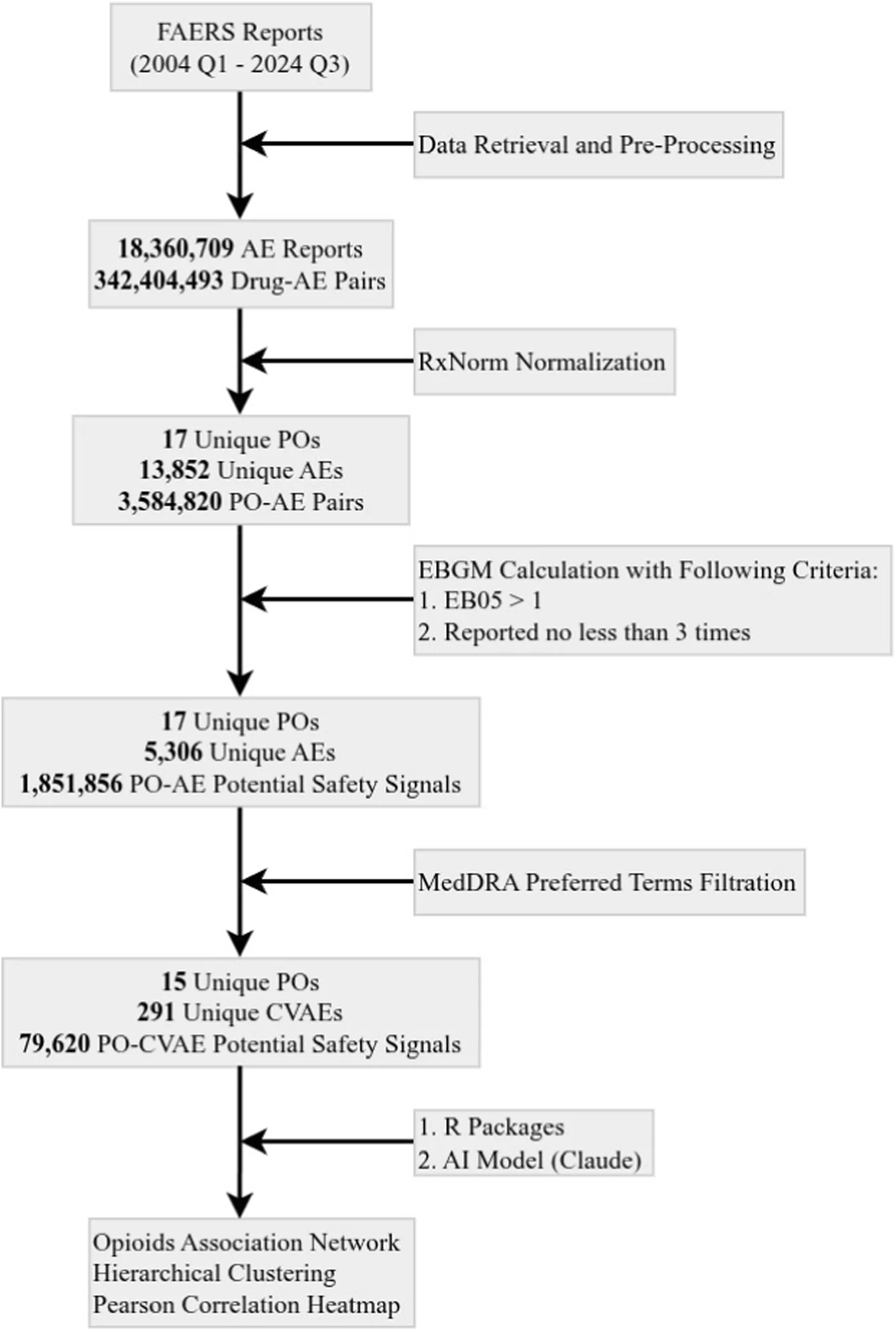
Data processing workflow for identifying CVAEs associated with POs using FAERS.

**TABLE 1:** Overall CVAE statistics for 17 FDA-approved opioid groups. For each opioid, five items were presented: 1) the count of reported PO-CVAE pairs, 2) the count of unique CVAEs, 3) the count of SDRs (Signals of Disproportionate Reporting), and 4) the ratios of SDRs to reported PO-CVAE pairs.

Category	Prescription opioid	PO-CVAE pairs (%)	Unique CVAEs	SDRs	Unique CVAEs of SDRs	SDR-CVAE ratio (%)
Natural	Codeine	11,117 (6.81%)	253	4,920	26	44.26
	Morphine	25,138 (15.39%)	397	16,317	104	64.91
Semi-synthetic	Hydrocodone	10,426 (6.38%)	297	5,272	56	50.57
	Hydromorphone	13,167 (8.06%)	286	6,234	26	47.35
	Oxycodone	26,474 (16.21%)	385	4,813	17	18.18
	Oxymorphone	518 (0.32%)	77	293	6	56.56
	Buprenorphine	3,911 (2.40%)	216	1,761	22	32.78
Synthetic	Butorphanol	46 (0.03%)	29	0	0	0.00
	Fentanyl	32,069 (19.63%)	411	22,866	117	71.30
	Levorphanol	16 (0.01%)	10	0	0	0.00
	Meperidine	823 (0.50%)	135	330	10	40.10
	Methadone	8,190 (5.02%)	273	3,779	44	46.14
	Pentazocine	580 (0.36%)	66	396	6	68.28
	Remifentanil	2,408 (1.47%)	156	2,175	64	90.32
	Sufentanil	2,421 (1.48%)	137	2,172	52	89.71
	Tapentadol	595 (0.36%)	89	130	8	21.85
	Tramadol	25,441 (15.57%)	399	8,641	44	33.96
	**Total**	**163,340 (100%)**	**539**	**79,620**	**291**	**48.74**

### 3.2 Overall statistics of PO-CVAEs

Table 1 lists the descriptive statistics of the PO-CVAE pairs in the dataset, categorizing the POs into groups of natural, semi-synthetic, and synthetic. It is noticed that 17 POs differed largely in the numbers of reported PO-CVAEs and SDRs. Fentanyl was the most-reported PO and had 32,069 PO-CVAE pairs (19.63% of the total) detected, followed by oxycodone (26,474, 16.21%), tramadol (25,441, 15.57%), morphine (25,138, 15.39%), etc., while only few of CVAEs were reported with levorphanol (16 PO-CVAEs pairs, 0.01% of the total) and butorphanol (46 PO-CVAEs pairs, 0.03% of the total). After disproportionality analysis, fentanyl still had the largest number of SDRs (22,866; 71.30% of its reported PO-CVAE pairs), including 117 unique CVAEs. Morphine (16,317), tramadol (8,641), and hydromorphone (6,234) followed. No SDRs were identified for butorphanol or levorphanol. It is noticeable that although the remifentanil-CVAE pairs only made up 1.47% of total reported pairs in the dataset, its SDR-CVAE ratio was the highest (90.32%). The same phenomena existed in sufentanil (89.71%) and pentazocine (68.28%), indicating the special patterns of the PO-CVAEs of these opioids.

### 3.3 Systematic comparison of CVAEs among POs

Table 2 presents the top 5 most reported CVAEs and calculated SDRs for each of the 15 POs (butorphanol and levorphanol are eliminated due to their limited reported CVAEs and no detected SDRs), including the PO-CVAE pair counts and proportions within each PO presented. This analysis aims to characterize the cardiovascular safety signals of all individual POs to inform clinicians and patients of potential CVAEs. Although there are certain similarities between the top 10 most reported CVAEs and top 10 SDRs for each of the POs (top 6–10 were not shown in the table due to the limited space), the top SDRs and the ranks are more meaningful for indicating potential safety signals. Each of the 15 POs was found to have several predominant SDRs detected (>10% of total SDRs). Many top 10 CVAEs SDRs are common among the 15 POs, and several CVAEs, including tachycardia, cardio-respiratory arrest, hypotension, cardiac arrest, and stress cardiomyopathy, etc., were frequently observed across most of the POs. Methadone showed a distinctive profile, with torsade de pointes ranking among its top 3 SDRs. Fentanyl, remifentanil, and sufentanil shared very similar SDR profiles and were associated with a disproportionately high number of hypotension, cardiac arrest, bradycardia, and tachycardia events. Pericarditis was a notably prominent SDR for oxycodone (43.9% of its SDRs) and codeine (19.0%).

**TABLE 2:** The top 5 most reported cardiovascular adverse events (CVAEs) and top 5 signals of disproportional reporting (SDRs) in each prescription opioid (PO).

Top 5 most reported CVAEs			Top 5 SDRs		
PT	Count	%	PT	Count	%
Buprenorphine					
Tachycardia	435	11.1	Tachycardia	435	33.9
Cardio-respiratory arrest	370	9.4	Cardio-respiratory arrest	370	28.9
Hypotension	229	5.8	Stress cardiomyopathy	90	7.0
Hypertension	225	5.7	Peripheral venous disease	79	6.2
Cardiac arrest	184	4.7	Cardiomegaly	52	4.1
Codeine					
Hypertension	1,802	16.2	Hypertension	1,800	36.6
Pericarditis	933	8.4	Pericarditis	933	19.0
Tachycardia	644	6.0	Palpitations	433	8.8
Hypotension	601	5.4	Peripheral venous disease	350	7.1
Palpitations	433	3.9	Pallor	289	5.9
Fentanyl					
Hypotension	3,486	10.9	Hypotension	3,486	15.2
Cardiac arrest	2,369	7.4	Cardiac arrest	2,369	10.4
Cardio-respiratory arrest	1,947	6.1	Cardio-respiratory arrest	1,947	8.5
Hypertension	1,902	5.9	Tachycardia	1,519	6.6
Tachycardia	1,519	4.7	Bradycardia	1,353	5.9
Hydrocodone					
Hypertension	842	8.1	Myocardial infarction	535	10.1
Hypotension	660	6.3	Atrial fibrillation	416	7.9
Myocardial infarction	535	5.1	Cardiac failure congestive	387	7.3
Cardiac arrest	509	4.9	Palpitations	371	7.0
Atrial fibrillation	416	4.0	Deep vein thrombosis	327	6.2
Hydromorphone					
Hypertension	1,291	9.8	Hypertension	1,291	20.7
Cardiogenic shock	1,263	9.6	Cardiogenic shock	1,263	20.3
Ventricular fibrillation	1,243	9.4	Ventricular fibrillation	1,243	19.9
Thrombosis	1,095	8.3	Thrombosis	1,095	17.6
Hypotension	813	6.2	Aortic stenosis	403	6.5
Meperidine					
Hypotension	126	15.3	Hypotension	126	38.2
Hypertension	83	10.1	Hypertension	83	25.2
Cardiac arrest	47	5.7	Deep vein thrombosis	35	10.6
Tachycardia	39	4.7	Bloody discharge	31	9.4
Deep vein thrombosis	35	4.3	Cardiac failure congestive	20	6.1
Methadone					
Cardiac arrest	1,023	12.5	Cardiac arrest	1,023	27.1
Cardio-respiratory arrest	722	8.8	Cardio-respiratory arrest	722	19.1
Hypotension	463	5.7	Torsade de pointes	362	9.6
Hypertension	459	5.6	Cardiac disorder	223	5.9
Tachycardia	383	4.7	Ventricular tachycardia	169	4.5
Morphine					
Hypotension	2,667	10.6	Hypotension	2,667	16.3
Hypertension	1,660	6.6	Cardiac arrest	1,442	8.8
Cardiac arrest	1,442	5.7	Tachycardia	1,112	6.8
Tachycardia	1,112	4.4	Myocardial infarction	776	4.8
Cardio-respiratory arrest	1,020	4.1	Bradycardia	769	4.7
Oxycodone					
Hypertension	2,767	10.4	Pericarditis	2,115	43.9
Pericarditis	2,115	8.0	Deep vein thrombosis	1,088	22.6
Hypotension	1,820	6.9	Peripheral venous disease	655	13.6
Cardiac arrest	1,360	5.1	Tricuspid valve incompetence	257	5.3
Deep vein thrombosis	1,088	4.1	Diastolic dysfunction	174	3.6
Oxymorphone					
Cardiac arrest	147	28.4	Cardiac arrest	147	50.2
Cardio-respiratory arrest	89	17.2	Cardio-respiratory arrest	89	30.4
Hypertension	20	3.9	Cardiomegaly	18	6.1
Cardiomegaly	18	3.5	Arteriosclerosis coronary artery	17	5.8
Ateriosclerosis coronary artery	17	3.3	Arteriosclerosis	14	4.8
Pentazocine					
Tachycardia	189	32.6	Tachycardia	189	47.7
Cardio-respiratory arrest	168	29.0	Cardio-respiratory arrest	168	42.4
Cardiac arrest	29	5.0	Circulatory collapse	14	3.5
Circulatory collapse	14	2.4	Arteriospasm coronary	12	3.0
Bradycardia	13	2.2	Ventricular extrasystoles	7	1.8
Remifentanil					
Hypotension	387	16.1	Hypotension	387	17.8
Cardiac arrest	261	10.8	Cardiac arrest	261	12.0
Bradycardia	259	10.8	Bradycardia	259	11.9
Tachycardia	130	5.4	Tachycardia	130	6.0
Hypertension	90	3.7	Hypertension	90	4.1
Sufentanil					
Hypotension	441	18.2	Hypotension	441	20.3
Tachycardia	215	8.9	Tachycardia	215	9.9
Cardiac arrest	194	8.0	Cardiac arrest	194	8.9
Cardio-respiratory arrest	134	5.5	Cardio-respiratory arrest	134	6.2
Bradycardia	116	4.8	Bradycardia	116	5.3
Tapentadol					
Hypertension	55	9.2	Hypertensive crisis	22	16.9
Hypotension	49	8.2	Cardiac failure	21	16.2
Cardiac arrest	39	6.6	Flushing	20	15.4
Tachycardia	32	5.4	Pallor	16	12.3
Atrial fibrillation	22	3.7	Bundle branch block left	15	11.5
Tramadol					
Hypertension	2,364	9.3	Cardiac disorder	1,135	13.1
Hypotension	2,031	8.0	Atrial fibrillation	947	11.0
Tachycardia	1,291	5.1	Palpitations	837	9.7
Cardiac disorder	1,135	4.5	Cardiac failure	606	7.0
Atrial fibrillation	947	3.7	Cardiac failure congestive	578	6.7

The top 5 CVAEs in the detected SDRs were calculated and the results were shown in Table 3. Hypotension (7,107 occurrences) was the most frequently identified SDR, followed by cardiac arrest (5,436), tachycardia (3,600), cardio-respiratory arrest (3,480), and hypertension (3,358). It was observed that hypotension and cardiac arrest were much more reported compared to tachycardia, cardio-respiratory arrest, and hypertension. The five pie charts in Figure 2 present the most contributing POs to each of the top 5 CVAEs identified in Table 3. 11 of the 15 POs shown in Figure 2 are contributors to the five CVAEs, while the rest of the four are not, therefore not included in the figure. Each chart represents a distinct CVAE, with numbers indicating the quantities of SDRs detected from each opioid. In the chart depicting hypertension, codeine and hydromorphone accounted for the largest share of SDRs. Fentanyl and morphine were the primary contributors to hypotension, cardiac arrest and tachycardia, together representing over 70% of each CVAE. Fentanyl was identified as a major contributor in 4 of the 5 CVAEs, including hypotension (3,486), cardiac arrest (2,369), cardio-respiratory arrest (1,947), and tachycardia (1,519), highlighting its potent cardiovascular risk profile among all prescription opioids examined. Notably, although remifentanil and sufentanil were not among the dominant contributors to any individual CVAE, both opioids appeared consistently across all five CVAEs, suggesting their broad cardiovascular risk profiles; therefore, cautious consideration should be used despite their relatively lower reporting volumes.

**TABLE 3:** Top 5 adverse events in detected SDRs.

Adverse events	Occur time
Hypotension	7,107
Cardiac arrest	5,436
Tachycardia	3,600
Cardio-respiratory arrest	3,480
Hypertension	3,358

**FIGURE 2:**
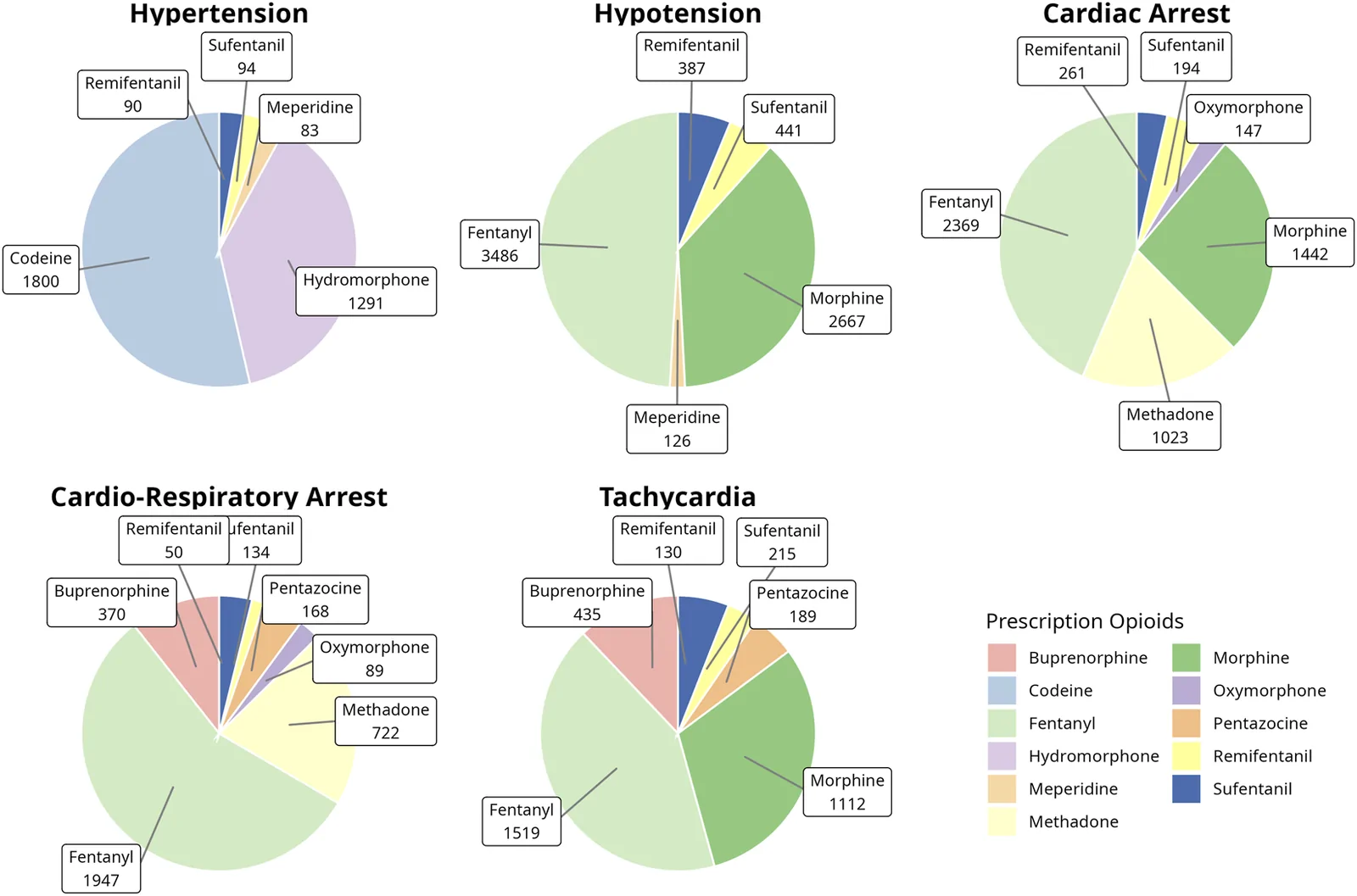
POs Contributions to the top five SDR CVAEs. Each pie chart represents a CVAE and exhibits the top POs contributors and their frequencies.

### 3.4 Analysis of PO relationships based on their CVAEs profiles using unsupervised machine learning

To identify the latent relationships among the 15 POs based on their potential safety signals of CVAEs, we applied unsupervised machine learning approaches. Network analysis was first applied on the SDRs of PO-CVAEs using the same methods in our previous work (Zhao et al., 2021; Le et al., 2023). In Figure 3, the nodes represent the 15 POs, with blue for synthetic opioids, green for natural opioids, and pink for the semi-synthetic opioids. The numbers in the nodes indicate the total shared CVAEs with other opioids. The numbers on the interconnecting lines between any two POs show the numbers of the shared CVAEs, with larger numbers corresponding to thicker lines. It was observed that fentanyl (206), morphine (193), remifentanil (171), sufentanil (139), hydrocodone (92), and methadone (87) have the biggest numbers and the thickest lines connecting each other, indicating significant numbers of shared CVAEs with other POs. The strongest associations were found between fentanyl and morphine with 51 shared CVAEs and between fentanyl and remifentanil with 48 shared CVAEs. There are 31 and 34 shared CVAEs between morphine and remifentanil, fentanyl and sufentanil, respectively. Interestingly, although having 4,813 identified SDRs (Table 1), oxycodone had the fewest shared CVAEs among the 15 POs (16). A similar pattern was observed for hydromorphone, which had the second fewest shared CVAEs (21) despite 6,234 detected SDRs (Table 1), suggesting that both opioids are associated with a more distinct and less overlapping set of cardiovascular signals. By contrast, pentazocine had 26 shared CVAEs despite only 396 SDRs. All the shared CVAEs between any two POs are listed in Supplementary Table S1.

**FIGURE 3:**
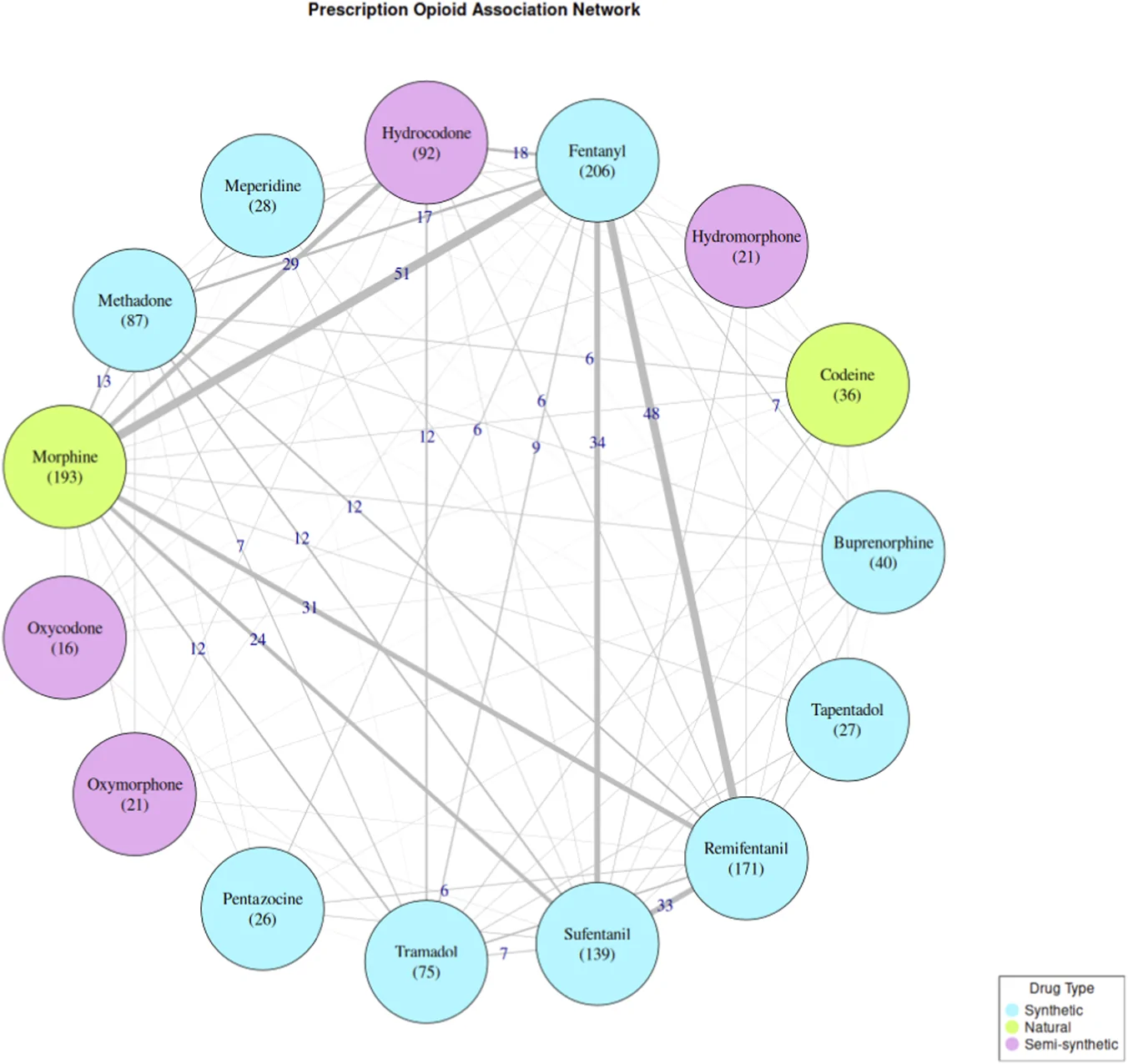
PO association network based on co-occurrence of SDR CVAEs. Each node represents a unique PO, with the number in parentheses indicating the total number of SDR CVAEs shared with other POs. The edges (lines) between nodes represent the number of shared SDR CVAEs; thicker lines indicate stronger co-occurrence relationships. Nodes are color-coded by opioid drug type, with green for natural opioids, purple for semi-synthetic opioids, and blue for synthetic opioids.

To further identify the relationships among the 15 POs based on their whole CVAE profiles, hierarchical clustering analysis was performed using a binary PO-CVAE matrix representing the presence or absence of each CVAE in the calculated SDR dataset. The results are shown as a dendrogram in Figure 4, where POs with similar CVAE presence/absence profiles are clustered together. The 15 POs are roughly divided into two major clusters at the highest level. Cluster A comprises only two POs, hydromorphone and oxycodone, which share a highly similar, but distinctly different CVAE presence/absence profile compared to all other opioids, merging in Cluster B only at the highest dissimilarity level. Cluster B comprises the remaining 13 POs. Within Cluster B, fentanyl, remifentanil, and sufentanil are grouped most closely together, with remifentanil and sufentanil forming the tightest pair before joining fentanyl and then morphine, reflecting their highly similar CVAE SDR profiles. Hydrocodone and tramadol also cluster closely. Codeine and tapentadol form their own sub-pair within Cluster B, along with buprenorphine, oxymorphone, and pentazocine. The hierarchical clustering analysis provided a complementary perspective to the network analysis and reinforced the observed associations among POs based on their full CVAEs profiles including presence or absence of CVAEs.

**FIGURE 4:**
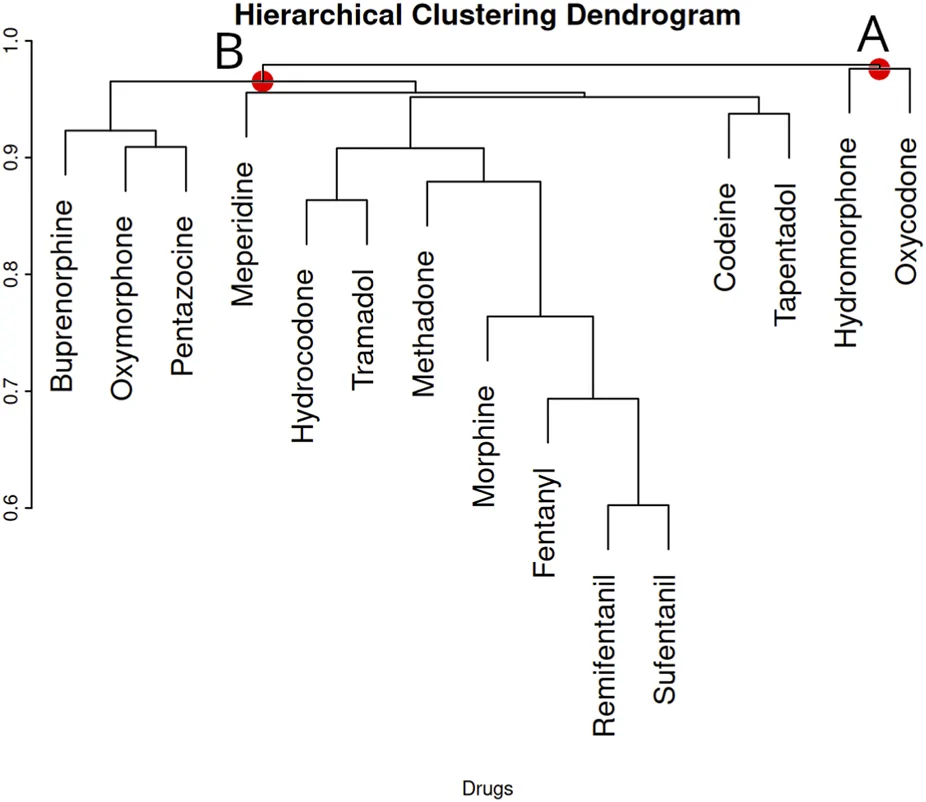
Hierarchical clustering dendrogram of POs based on the whole profiles including presence and absence of SDR CVAEs.

### 3.5 Pearson correlation analysis reveals distinct cardiovascular risk clusters among prescription opioids

To complement the network analysis and hierarchical clustering analyses, a Pearson correlation matrix was computed to quantify pairwise similarities among the 15 POs based on their SDR CVAE profiles with the assistance of Claude. The results are presented as a clustered heatmap in Figure 5, showing various correlations among the 15 POs. Several notable high-correlation clusters were identified. Fentanyl, remifentanil, and sufentanil, which belong to synthetic opioids, formed the highest correlation cluster (r = 0.89–0.91), reflecting their shared top 10 CVAEs in the detected SDRs identified in Table 2. These three synthetic opioids also showed high correlations with the natural opioid morphine, and the results were consistent with those of network analysis and hierarchical cluster analysis (Figures 3, 4). Strong pairwise correlations were also observed between oxymorphone and methadone (r = 0.92) and between pentazocine and buprenorphine (r = 0.96), consistent with their shared SDR profiles in Table 2. In contrast, hydromorphone showed limited correlation with most POs despite having a substantial number of reported CVAEs and SDRs (Table 1), confirming its distinct cardiovascular profile dominated by cardiogenic shock and ventricular fibrillation (Table 2). Tapentadol and meperidine were largely uncorrelated with the remaining POs, consistent with their sparse or distinct CVAE SDR profiles. The hierarchical clustering order in the heatmap was fully consistent with the dendrogram in Figure 4, with morphine-fentanyl-remifentanil-sufentanil confirmed as the most internally coherent cluster in the dataset.

**FIGURE 5:**
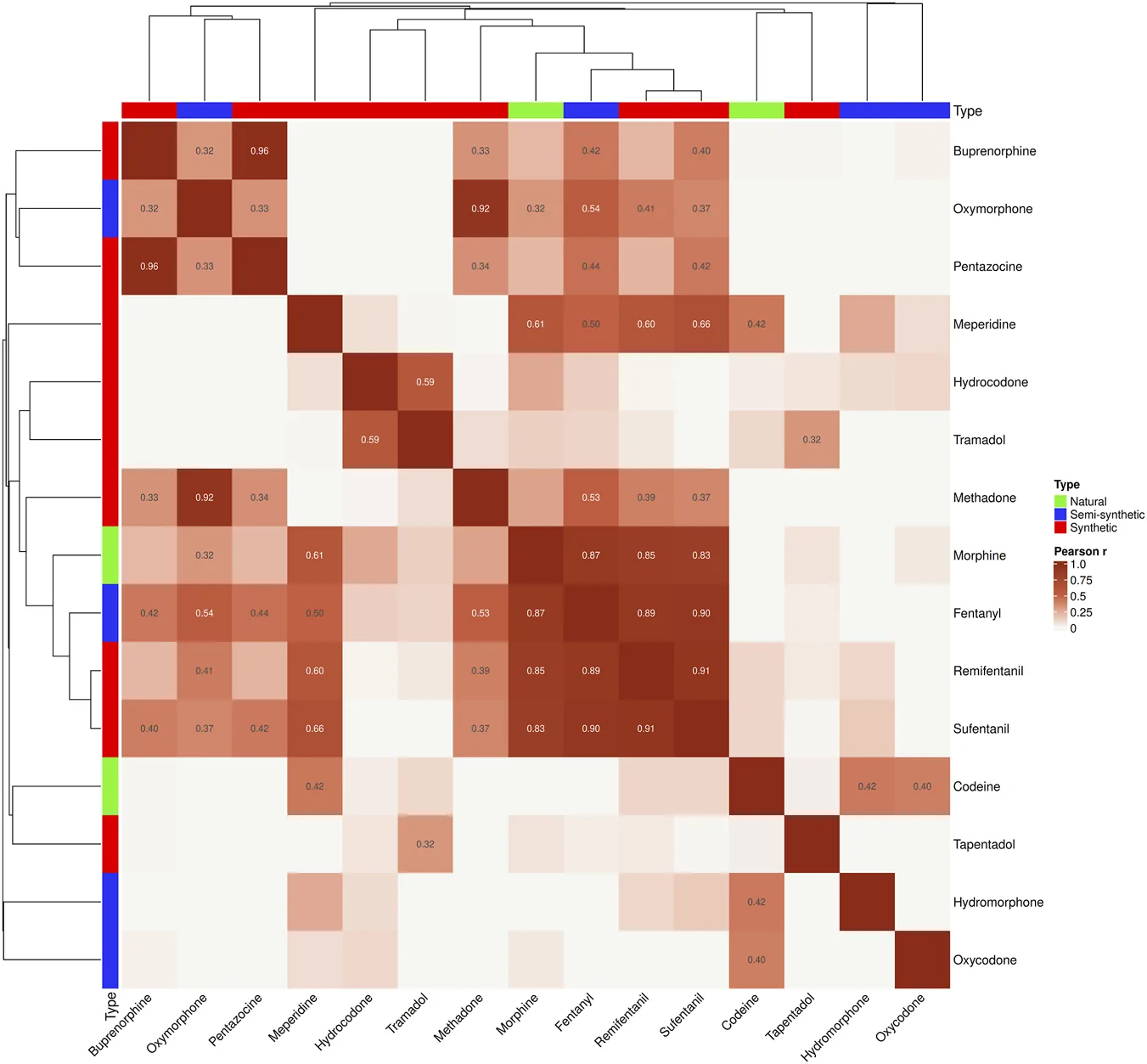
Pearson Correlation Heatmap of POs based on shared SDR CVAEs.

## 4 Discussion

Cardiovascular complications are among the most serious and life-threatening AEs, particularly in patients with existing heart disease or those using other cardiotoxic agents. POs are widely used for pain management but are increasingly recognized for their association with serious cardiovascular risks. It was indicated that even short-term opioid use can lead to adverse cardiovascular events, with higher doses and longer durations associated with increased risk (Chen and Ashburn, 2015). To the best of our knowledge, this study is the first to systematically mine the FAERS database specifically for CVAEs associated with widely used POs, providing a focused and comparative assessment of their cardiac safety profiles.

In this study, a big dataset has been built up starting from over 18 million reports and 342 million drug-AE pairs from the FAERS database. Among the potential safety signals or SDRs after disproportionality analysis, 79,620 (4.3%) were associated with CVDAEs, highlighting significant associations between specific POs and cardiovascular adverse events. The big size of the dataset and the workflow for the data pre-processing and mining have been used to minimize the reporting bias of FAERS data. The observed distribution of PO-CVAE pairs across the 17 POs (Table 1) reflected the different levels of cardiovascular risks, showing the necessities in understanding potential cardiovascular-related adverse events of different POs. While milder events such as hot flush, pallor and cyanosis etc. were also observed, the presence of serious CVAEs, such as cardiac arrest, myocardial infarction, and arrhythmia, etc. (Table 2), highlights the importance of CVAE assessment prior to initiating opioid therapy. The detected top 5 CVAE (Table 3) and the main contributing POs (Figure 2) provide important systematic information for both physicians and patients when prescribing and using opioids and emphasized the need for PO-specific cardiovascular risk assessment in clinical decision-making.

Fentanyl, oxycodone, tramadol and morphine were revealed to have the highest number of reported PO-CVAEs and SDRs, reflecting both their pharmacological effects and widespread clinical use; while levorphanol and butorphanol have the lowest PO-CVAEs reported compared to the other 15 POs, which may be due to their limited clinical use and low CVAE risk profiles. Although some overlapping of CVAEs was observed among the top 10 SDRs of the 15 POs (top 6–10 not shown in the Table 2), the top several (1–4) were much more frequent than the others. For example, tachycardia and cardio-respiratory arrest are the top 2 CVAEs for buprenorphine and the percentages of their SDRs are much higher than the others. If we take 25% as the cut-off value, tachycardia and cardio-respiratory arrest can be considered as the signature marker CVAEs for buprenorphine (Table 2). Therefore, the CVAE markers are potentially available as the risk labels of POs for the cardiovascular safety patterns.

The results in Tables 1–3; Figures 2–5 are highly consistent with each other and systematically clarify the cardiovascular safety patterns of the 17 POs in different aspects. Fentanyl was most reported with hypotension, cardiac arrest, cardio-respiratory arrest and tachycardia SDRs, which were four of the top five most-reported SDR-CVAEs (Figure 2; Table 2). Its cardiovascular safety pattern was found very similar to remifentanil and sufentanil (Figure 2; Table 2). The three POs shared many common CVAEs (Figure 3) and clustered together due to their similar CVAE profiles (Figures 4, 5). Given that fentanyl, remifentanil, and sufentanil are all synthetic opioids in the phenylpiperidine class, this interesting observation may reflect their shared structural class and pharmacologic properties. All three synthetic opioids showed similar associations with hypotension, bradycardia, cardio-respiratory arrest, tachycardia, and cardiac arrest, and is consistent with their potent μ-opioid receptor agonism and clinical use in high-acuity settings (Engoren et al., 2001; Myles et al., 2002). The remaining one of the top five most-reported SDR-CVAEs was hypertension, which was mostly reported with codeine and hydromorphone. Interestingly, hydromorphone and codeine differ substantially in chemical structure and mechanism of action. Hydromorphone is a semisynthetic opioid structurally related to morphine, while codeine is a naturally derived opioid. This unexpected result may warrant exploration in future work.

Several factors may contribute to the observed opioid clusters other than shared receptor pharmacology. The tight fentanyl-remifentanil-sufentanil-morphine cluster is consistent with their common use in perioperative and critical-care settings, where hemodynamic events such as hypotension, bradycardia, and cardiac arrest are frequently observed and reported (Chow et al., 2021; Krantz et al., 2021). The resulting associations may therefore partly reflect confounding by shared reporting context rather than intrinsic drug effects. Similarly, correlations among agents such as oxymorphone, methadone, pentazocine, and buprenorphine may arise from overlapping patient populations and prescribing patterns as much as from pharmacological similarity (Coffa and Snyder, 2019; Cutroneo et al., 2023; Lei et al., 2025; Kaya and Yorulmaz Kaya, 2026). Disentangling these explanations will require analyses that account for indication, dose, route, and co-reported medications, which spontaneous reporting data alone cannot fully support.

Notably, when the network analysis, hierarchical clustering analyses, and Pearson correlation analysis were applied exclusively to SDR CVAEs signals in this study, the resulting PO relations/associations differed from those generated using whole AE profiles in our previous work (Le et al., 2023). Whole AE profiles included diverse events, while CVAEs covered only cardiac-specific risks. Some POs with many shared general AE profiles (e.g., fentanyl and hydromorphone) (Le et al., 2023) may differ significantly in CVAE-based patterns (this study). Fentanyl-hydromorphone and morphine-hydrocodone were found to have strong associations in the network analysis by sharing 28 whole AEs, and closely clustered together in the hierarchical analysis by the similar presence/absence whole AE profiles (Le et al., 2023); while the two pairs of POs have few shared CVAEs and showed a distance in the clusters when the analysis was based on the SDR CVAEs in this study (Figures 3–5). The top 10 CVAEs of these two POs were found to have no overlapping. Moreover, the results of network analysis seemed to have more changes than the hierarchical cluster analysis when limited to SDR CVAEs, as this method relies on the number and type of CVAEs shared between POs to construct shared CVAE-based relationships. Filtering to CVAEs highlighted new inter-drug connections specific to cardiovascular risks that were previously obscured by the broader AE profile in the previous work. In contrast, the hierarchical clustering results showed less differences relatively. This is likely due to its use of the full presence/absence pattern across CVAEs for all POs, which still retained certain similarities to the full AE matrix. The comparison with the previous studies shows differences which may indicate the importance of condition-focused pharmacovigilance, and the organ-specific safety analyses are expected to uncover hidden risk relationships that may be masked in broader assessments.

This study further illustrates how machine learning-enabled pharmacovigilance can enhance organ-specific drug safety evaluation. By integrating empirical Bayes signal detection with unsupervised network and clustering methods, we uncovered latent cardiovascular risk patterns and opioid groupings that were not apparent from whole adverse-event profiles alone. Such AI-assisted analyses support more nuanced, mechanism-informed safety assessments and highlight the potential role of interpretable machine learning approaches in regulatory decision-making and post-marketing surveillance.

This study has several limitations inhered from the use of FAERS. FAERS is a passive surveillance system subject to the following facts, including but not limited to substantial reporting bias, underreporting, and varied reporting quality, etc. Since the database lacks a defined denominator of exposed patients, disproportionality metrics such as EBGM quantify relative reporting and cannot be used to estimate incidence, absolute risk, or causality. The analyses also cannot adjust for important confounding factors, such as comorbidities, concomitant medications, dose, route, and duration of therapy, etc. All these factors are particularly relevant for opioids used in high-acuity settings. Duplicate reports from the same reporters cannot be eliminated, although the FDA-recommended deduplication was already performed in this study. The big size of the dataset (18 M) and the workflow for the data pre-processing and mining were expected to minimize the reporting bias of FAERS data.

In conclusion, this study was strengthened using a large national database and the integration of data mining and complementary analytical approaches. Compared with prior whole-AE profile studies, our CVAE-focused analysis offers more precise insight into the cardiovascular risks posed by individual opioids. The findings highlighted the need for careful risk assessment and monitoring, such as electrocardiograms (ECGs) screening with methadone or cardiovascular monitoring during fentanyl administration. The results support more refined prescribing practices and may help optimize opioid safety labeling and regulatory decision-making to mitigate prescription opioid-related cardiovascular harm.

## Data Availability

All data are available at https://fis.fda.gov/extensions/FPD-QDE-FAERS/FPD-QDE-FAERS.html.
