# Supplementary material for: Two decades of prescription opioid-related cardiovascular safety signals: a FAERS-based pharmacovigilance study using machine learning
Source: Front Drug Saf Regul. 2026 Sep 28;6:1898403. doi: 10.3389/fdsfr.2026.1898403 (PMC13655235; doi:10.3389/fdsfr.2026.1898403)
Supplement: Supplementary file 1 [file Table1.docx]

# 1. Supplementary Table 1

| **Prescription Opioid Pair** | **Shared Cardiovascular Adverse Events Count** | **Shared Cardiovascular Adverse Events** |
| --- | --- | --- |
| Fentanyl-Morphine | 51 | acute myocardial infarction,aortic arteriosclerosis,aortic valve incompetence,atrial fibrillation,atrioventricular dissociation,bradycardia,bradycardia neonatal,bundle branch block left,cardiac arrest,cardiac failure,cardiac failure acute,cardiac tamponade,cardiopulmonary failure,cardiotoxicity,cardiovascular disorder,coronary artery disease,coronary artery thrombosis,dilatation atrial,dilatation ventricular,extrasystoles,haematoma,haemodynamic instability,haemorrhage,hypoperfusion,hypotension,hypovolaemic shock,left ventricular hypertrophy,mitral valve incompetence,myocardial infarction,myocardial ischaemia,myocarditis,nodal rhythm,pericardial effusion,peripheral ischaemia,peripheral vascular disorder,pulseless electrical activity,restrictive cardiomyopathy,rhythm idioventricular,right ventricular failure,shock,shock haemorrhagic,sinus bradycardia,sinus tachycardia,supraventricular extrasystoles,supraventricular tachycardia,tachyarrhythmia,tachycardia,tricuspid valve incompetence,ventricular dysfunction,ventricular extrasystoles,ventricular tachycardia |
| Fentanyl-Remifentanil | 48 | acute coronary syndrome,air embolism,arrhythmia,arteriospasm coronary,atrial fibrillation,atrioventricular block,atrioventricular block complete,atrioventricular block second degree,atrioventricular dissociation,bradycardia,bradycardia foetal,bundle branch block left,bundle branch block right,cardiac arrest,cardiac failure,cardiac failure acute,cardio-respiratory arrest,cardiopulmonary failure,cardiovascular disorder,circulatory collapse,cyanosis,haematoma,haemodynamic instability,haemorrhage,hypertensive crisis,hypotension,hypovolaemic shock,kounis syndrome,mitral valve incompetence,myocardial ischaemia,nodal rhythm,pericardial effusion,peripheral ischaemia,prinzmetal angina,pulseless electrical activity,rhythm idioventricular,shock,shock haemorrhagic,sinus arrest,sinus bradycardia,sinus tachycardia,stress cardiomyopathy,supraventricular extrasystoles,supraventricular tachycardia,tachyarrhythmia,tachycardia,ventricular extrasystoles,ventricular tachycardia |
| Fentanyl-Sufentanil | 34 | acute myocardial infarction,arrhythmia,atrioventricular block,atrioventricular block complete,bradycardia,bradycardia foetal,cardiac arrest,cardiac failure,cardiac failure acute,cardio-respiratory arrest,cardiovascular disorder,circulatory collapse,cyanosis,haematoma,haemodynamic instability,haemorrhage,hyperdynamic left ventricle,hypertensive crisis,hypotension,long qt syndrome,myocardial ischaemia,pulseless electrical activity,right ventricular dysfunction,shock,shock haemorrhagic,sinus bradycardia,sinus tachycardia,stress cardiomyopathy,supraventricular tachycardia,tachycardia,ventricular arrhythmia,ventricular extrasystoles,ventricular hypokinesia,ventricular tachycardia |
| Remifentanil-Sufentanil | 33 | arrhythmia,atrioventricular block,atrioventricular block complete,bradycardia,bradycardia foetal,cardiac arrest,cardiac failure,cardiac failure acute,cardio-respiratory arrest,cardiogenic shock,cardiovascular disorder,circulatory collapse,cyanosis,haematoma,haemodynamic instability,haemorrhage,hypertension,hypertensive crisis,hypotension,myocardial ischaemia,pericardial haemorrhage,pulseless electrical activity,shock,shock haemorrhagic,sinus bradycardia,sinus tachycardia,stress cardiomyopathy,supraventricular tachycardia,tachycardia,torsade de pointes,ventricular extrasystoles,ventricular fibrillation,ventricular tachycardia |
| Morphine-Remifentanil | 31 | atrial fibrillation,atrioventricular dissociation,bradycardia,bundle branch block left,cardiac arrest,cardiac failure,cardiac failure acute,cardiopulmonary failure,cardiovascular disorder,haematoma,haemodynamic instability,haemorrhage,hypotension,hypovolaemic shock,mitral valve incompetence,myocardial ischaemia,nodal rhythm,pericardial effusion,peripheral ischaemia,pulseless electrical activity,rhythm idioventricular,shock,shock haemorrhagic,sinus bradycardia,sinus tachycardia,supraventricular extrasystoles,supraventricular tachycardia,tachyarrhythmia,tachycardia,ventricular extrasystoles,ventricular tachycardia |
| Hydrocodone-Morphine | 29 | aneurysm,angina pectoris,aortic arteriosclerosis,aortic valve incompetence,arterial occlusive disease,arteriosclerosis,atrial fibrillation,atrial tachycardia,cardiac failure congestive,cardiac valve disease,cardiomegaly,cardiovascular disorder,coronary artery disease,deep vein thrombosis,dilatation atrial,dilatation ventricular,haemorrhage,ischaemic cardiomyopathy,left atrial dilatation,left ventricular hypertrophy,mitral valve disease,mitral valve incompetence,myocardial infarction,peripheral arterial occlusive disease,peripheral coldness,peripheral vascular disorder,poor peripheral circulation,supraventricular extrasystoles,tricuspid valve incompetence |
| Morphine-Sufentanil | 24 | acute myocardial infarction,angiopathy,bradycardia,capillary leak syndrome,cardiac arrest,cardiac failure,cardiac failure acute,cardiovascular disorder,haematoma,haemodynamic instability,haemorrhage,hypotension,ischaemia,left ventricular failure,myocardial ischaemia,pulseless electrical activity,shock,shock haemorrhagic,sinus bradycardia,sinus tachycardia,supraventricular tachycardia,tachycardia,ventricular extrasystoles,ventricular tachycardia |
| Fentanyl-Hydrocodone | 18 | aortic arteriosclerosis,aortic valve incompetence,atrial fibrillation,atrial flutter,cardiovascular disorder,coronary artery disease,dilatation atrial,dilatation ventricular,haemorrhage,hot flush,left ventricular hypertrophy,mitral valve incompetence,myocardial infarction,peripheral vascular disorder,pulmonary valve incompetence,supraventricular extrasystoles,tricuspid valve incompetence,vein disorder |
| Fentanyl-Methadone | 17 | arrhythmia,cardiac arrest,cardiac failure acute,cardio-respiratory arrest,cardiopulmonary failure,cardiotoxicity,cyanosis,left ventricular hypertrophy,long qt syndrome,sinus bradycardia,stress cardiomyopathy,supraventricular extrasystoles,vasodilatation,ventricular arrhythmia,ventricular extrasystoles,ventricular tachycardia,withdrawal hypertension |
| Methadone-Morphine | 13 | arterial disorder,arteriosclerosis coronary artery,cardiac arrest,cardiac failure acute,cardiomegaly,cardiopulmonary failure,cardiotoxicity,left ventricular hypertrophy,mitral valve disease,sinus bradycardia,supraventricular extrasystoles,ventricular extrasystoles,ventricular tachycardia |
| Hydrocodone-Tramadol | 12 | aneurysm,atrial fibrillation,cardiac disorder,cardiac failure congestive,cardiac flutter,coronary artery disease,coronary artery occlusion,flushing,hot flush,internal haemorrhage,palpitations,peripheral coldness |
| Methadone-Remifentanil | 12 | arrhythmia,cardiac arrest,cardiac failure acute,cardio-respiratory arrest,cardiopulmonary failure,cyanosis,sinus bradycardia,stress cardiomyopathy,supraventricular extrasystoles,torsade de pointes,ventricular extrasystoles,ventricular tachycardia |
| Methadone-Sufentanil | 12 | arrhythmia,cardiac arrest,cardiac failure acute,cardio-respiratory arrest,cyanosis,long qt syndrome,sinus bradycardia,stress cardiomyopathy,torsade de pointes,ventricular arrhythmia,ventricular extrasystoles,ventricular tachycardia |
| Morphine-Tramadol | 12 | aneurysm,atrial fibrillation,cardiac failure,cardiac failure acute,cardiac failure congestive,coronary artery disease,haematoma,heart valve incompetence,orthostatic hypotension,pallor,peripheral coldness,phlebitis |
| Remifentanil-Tramadol | 11 | arrhythmia,atrial fibrillation,brash syndrome,cardiac failure,cardiac failure acute,circulatory collapse,cyanosis,flushing,haematoma,myocardial depression,pericardial haemorrhage |
| Fentanyl-Tramadol | 9 | arrhythmia,atrial fibrillation,cardiac failure,cardiac failure acute,circulatory collapse,coronary artery disease,cyanosis,haematoma,hot flush |
| Buprenorphine-Fentanyl | 7 | bradycardia foetal,cardio-respiratory arrest,foetal heart rate deceleration abnormality,peripheral ischaemia,stress cardiomyopathy,tachycardia,tachycardia foetal |
| Hydrocodone-Methadone | 7 | cardiac disorder,cardiomegaly,left ventricular hypertrophy,mitral valve disease,mitral valve prolapse,supraventricular extrasystoles,vascular occlusion |
| Meperidine-Morphine | 7 | arterial disorder,cardiac failure congestive,deep vein thrombosis,hypotension,restrictive cardiomyopathy,venoocclusive disease,ventricular tachycardia |
| Methadone-Tramadol | 7 | arrhythmia,atrioventricular block first degree,cardiac disorder,cardiac failure acute,cardiac failure chronic,cyanosis,myocardial fibrosis |
| Sufentanil-Tramadol | 7 | arrhythmia,cardiac failure,cardiac failure acute,circulatory collapse,cyanosis,haematoma,pericardial haemorrhage |
| Buprenorphine-Remifentanil | 6 | bradycardia foetal,cardio-respiratory arrest,myocardial depression,peripheral ischaemia,stress cardiomyopathy,tachycardia |
| Codeine-Methadone | 6 | arteriosclerosis coronary artery,cyanosis,left ventricular dysfunction,myocardial fibrosis,myocardial necrosis,systolic dysfunction |
| Fentanyl-Pentazocine | 6 | arteriospasm coronary,cardio-respiratory arrest,circulatory collapse,shock haemorrhagic,tachycardia,ventricular extrasystoles |
| Hydrocodone-Remifentanil | 6 | atrial fibrillation,cardiovascular disorder,flushing,haemorrhage,mitral valve incompetence,supraventricular extrasystoles |
| Pentazocine-Remifentanil | 6 | arteriospasm coronary,cardio-respiratory arrest,circulatory collapse,shock haemorrhagic,tachycardia,ventricular extrasystoles |
| Buprenorphine-Morphine | 5 | cardiomegaly,orthostatic hypotension,peripheral ischaemia,tachycardia,venous thrombosis |
| Codeine-Tramadol | 5 | arteriovenous fistula,cyanosis,myocardial fibrosis,pallor,palpitations |
| Hydromorphone-Sufentanil | 5 | cardiogenic shock,hypertension,ischaemia,ventricular fibrillation,ventricular hypokinesia |
| Pentazocine-Sufentanil | 5 | cardio-respiratory arrest,circulatory collapse,shock haemorrhagic,tachycardia,ventricular extrasystoles |
| Remifentanil-Tapentadol | 5 | arrhythmia,bundle branch block left,cardiac failure,flushing,hypertensive crisis |
| Tapentadol-Tramadol | 5 | arrhythmia,cardiac failure,flushing,orthostatic hypotension,pallor |
| Buprenorphine-Methadone | 4 | cardio-respiratory arrest,cardiomegaly,stress cardiomyopathy,visceral congestion |
| Buprenorphine-Sufentanil | 4 | bradycardia foetal,cardio-respiratory arrest,stress cardiomyopathy,tachycardia |
| Codeine-Morphine | 4 | angiopathy,arteriosclerosis coronary artery,pallor,poor peripheral circulation |
| Fentanyl-Meperidine | 4 | hypertensive crisis,hypotension,restrictive cardiomyopathy,ventricular tachycardia |
| Fentanyl-Tapentadol | 4 | arrhythmia,bundle branch block left,cardiac failure,hypertensive crisis |
| Hydromorphone-Remifentanil | 4 | cardiogenic shock,hypertension,sinus node dysfunction,ventricular fibrillation |
| Meperidine-Remifentanil | 4 | hypertension,hypertensive crisis,hypotension,ventricular tachycardia |
| Meperidine-Sufentanil | 4 | hypertension,hypertensive crisis,hypotension,ventricular tachycardia |
| Methadone-Oxymorphone | 4 | arteriosclerosis coronary artery,cardiac arrest,cardio-respiratory arrest,cardiomegaly |
| Morphine-Oxymorphone | 4 | arteriosclerosis,arteriosclerosis coronary artery,cardiac arrest,cardiomegaly |
| Morphine-Tapentadol | 4 | bundle branch block left,cardiac failure,orthostatic hypotension,pallor |
| Buprenorphine-Tramadol | 3 | femoral artery aneurysm,myocardial depression,orthostatic hypotension |
| Codeine-Fentanyl | 3 | cardiac dysfunction,cardiovascular insufficiency,cyanosis |
| Codeine-Hydrocodone | 3 | palpitations,poor peripheral circulation,raynaud's phenomenon |
| Codeine-Remifentanil | 3 | cyanosis,distributive shock,hypertension |
| Codeine-Sufentanil | 3 | angiopathy,cyanosis,hypertension |
| Hydrocodone-Hydromorphone | 3 | aortic aneurysm,thrombosis,varicose vein |
| Hydrocodone-Oxycodone | 3 | deep vein thrombosis,internal haemorrhage,tricuspid valve incompetence |
| Hydrocodone-Oxymorphone | 3 | arteriosclerosis,cardiomegaly,hypertensive heart disease |
| Hydrocodone-Sufentanil | 3 | cardiovascular disorder,conduction disorder,haemorrhage |
| Hydromorphone-Morphine | 3 | blood pressure fluctuation,ischaemia,vasculitis |
| Morphine-Oxycodone | 3 | deep vein thrombosis,embolism,tricuspid valve incompetence |
| Morphine-Pentazocine | 3 | shock haemorrhagic,tachycardia,ventricular extrasystoles |
| Sufentanil-Tapentadol | 3 | arrhythmia,cardiac failure,hypertensive crisis |
| Buprenorphine-Hydrocodone | 2 | cardiomegaly,varicose vein |
| Buprenorphine-Oxycodone | 2 | peripheral venous disease,tachycardia foetal |
| Buprenorphine-Oxymorphone | 2 | cardio-respiratory arrest,cardiomegaly |
| Buprenorphine-Pentazocine | 2 | cardio-respiratory arrest,tachycardia |
| Codeine-Hydromorphone | 2 | hypertension,lymphoedema |
| Codeine-Oxycodone | 2 | pericarditis,peripheral venous disease |
| Codeine-Tapentadol | 2 | pallor,subclavian vein thrombosis |
| Fentanyl-Oxycodone | 2 | tachycardia foetal,tricuspid valve incompetence |
| Fentanyl-Oxymorphone | 2 | cardiac arrest,cardio-respiratory arrest |
| Hydrocodone-Meperidine | 2 | cardiac failure congestive,deep vein thrombosis |
| Meperidine-Methadone | 2 | arterial disorder,ventricular tachycardia |
| Methadone-Pentazocine | 2 | cardio-respiratory arrest,ventricular extrasystoles |
| Oxycodone-Tramadol | 2 | giant cell arteritis,internal haemorrhage |
| Oxymorphone-Remifentanil | 2 | cardiac arrest,cardio-respiratory arrest |
| Oxymorphone-Sufentanil | 2 | cardiac arrest,cardio-respiratory arrest |
| Buprenorphine-Codeine | 1 | peripheral venous disease |
| Buprenorphine-Hydromorphone | 1 | varicose vein |
| Buprenorphine-Tapentadol | 1 | orthostatic hypotension |
| Codeine-Meperidine | 1 | hypertension |
| Codeine-Oxymorphone | 1 | arteriosclerosis coronary artery |
| Fentanyl-Hydromorphone | 1 | ventricular hypokinesia |
| Hydrocodone-Tapentadol | 1 | flushing |
| Hydromorphone-Meperidine | 1 | hypertension |
| Hydromorphone-Oxycodone | 1 | granulomatosis with polyangiitis |
| Meperidine-Oxycodone | 1 | deep vein thrombosis |
| Meperidine-Tapentadol | 1 | hypertensive crisis |
| Meperidine-Tramadol | 1 | cardiac failure congestive |
| Methadone-Tapentadol | 1 | arrhythmia |
| Oxymorphone-Pentazocine | 1 | cardio-respiratory arrest |
| Pentazocine-Tramadol | 1 | circulatory collapse |
